# Supplementary figures and images for: Evolutionary Responses to Acquiring a Multidrug Resistance Plasmid Are Dominated by Metabolic Functions across Diverse Escherichia coli Lineages
Source: mSystems. 2023 Feb 1;8(1):e00713-22. doi: 10.1128/msystems.00713-22 (PMC9948715; doi:10.1128/msystems.00713-22)

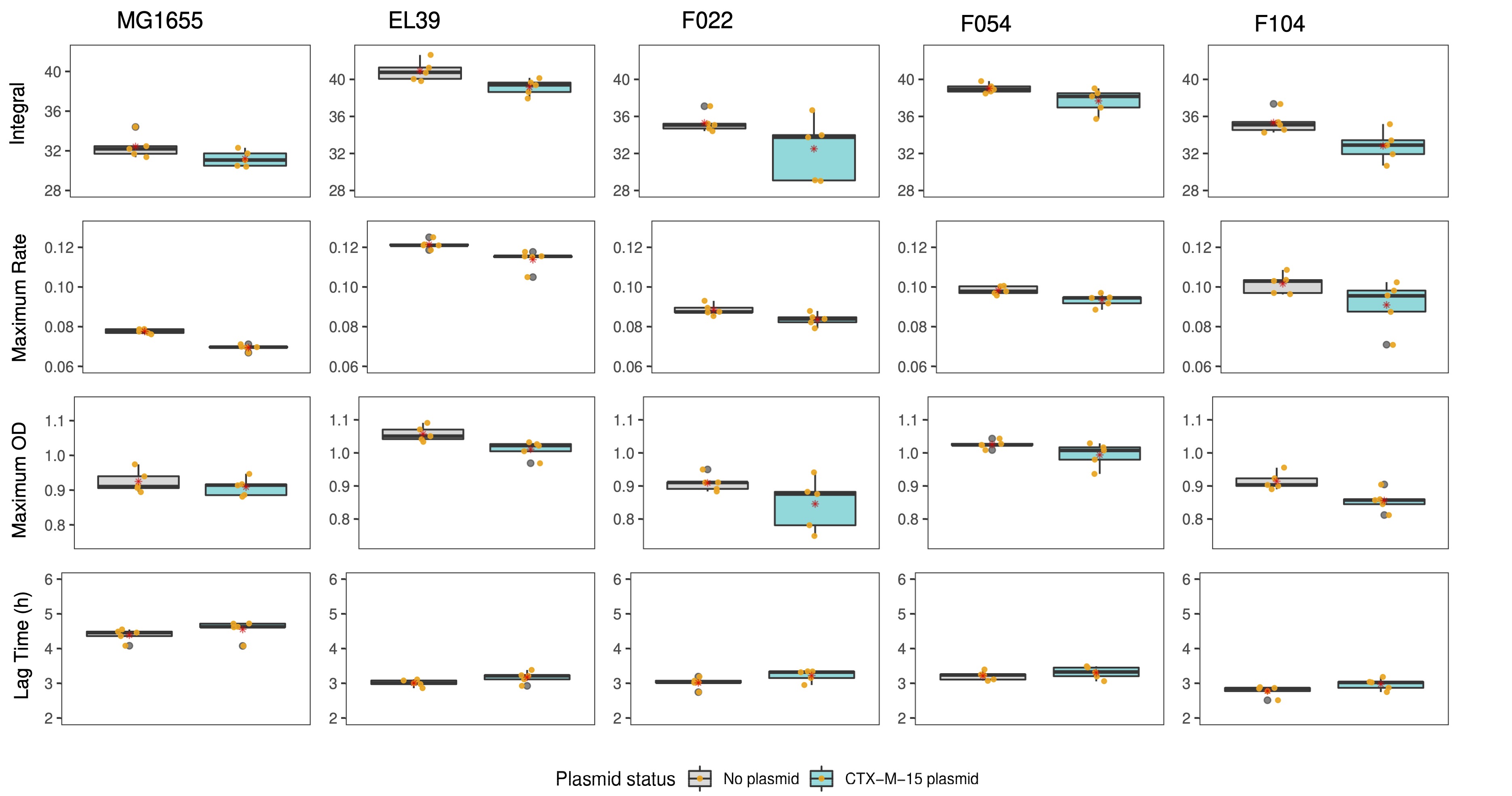

Supplement: FIG S1 [file msystems.00713-22-s0005.jpg]

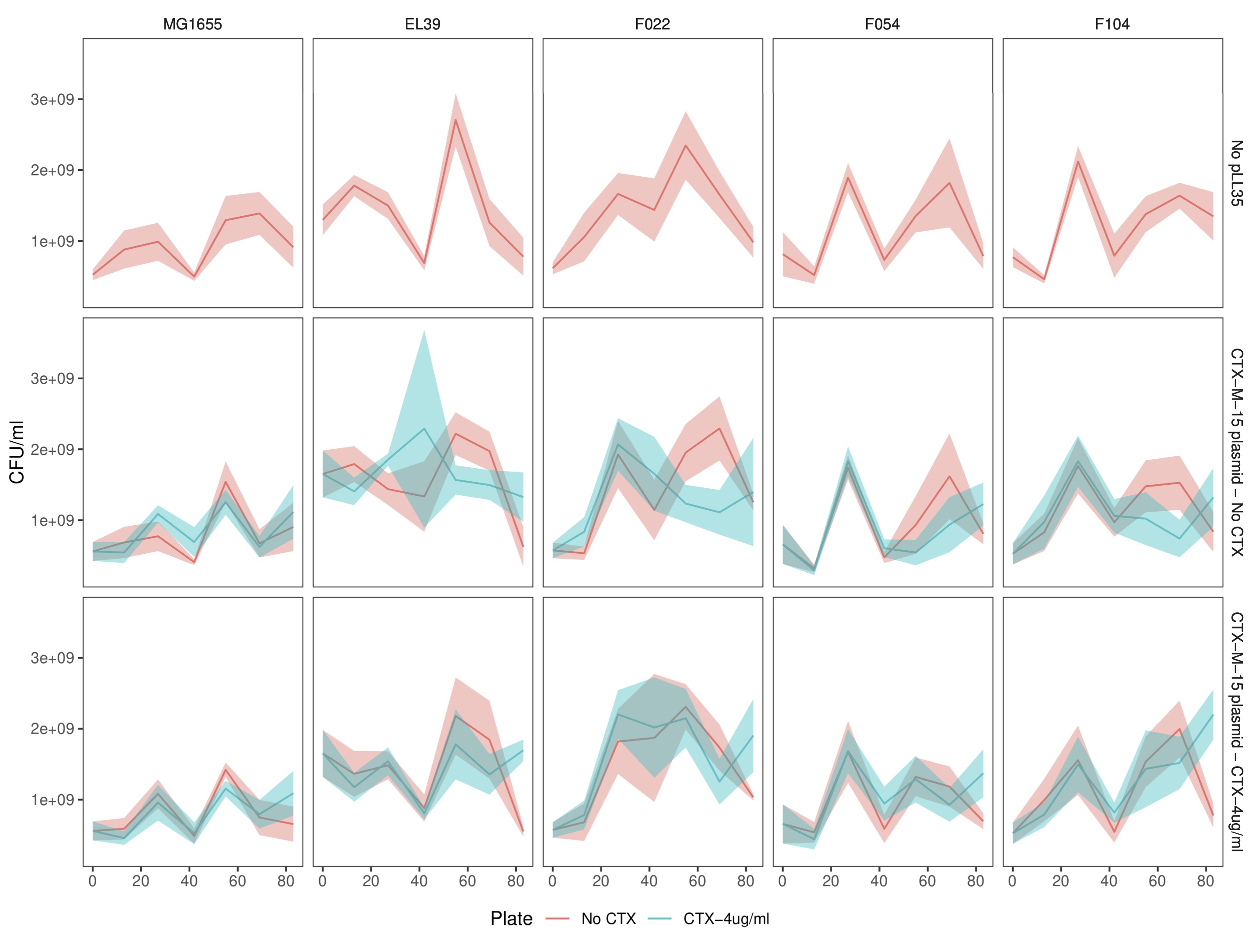

Supplement: FIG S2 [file msystems.00713-22-s0006.jpg]

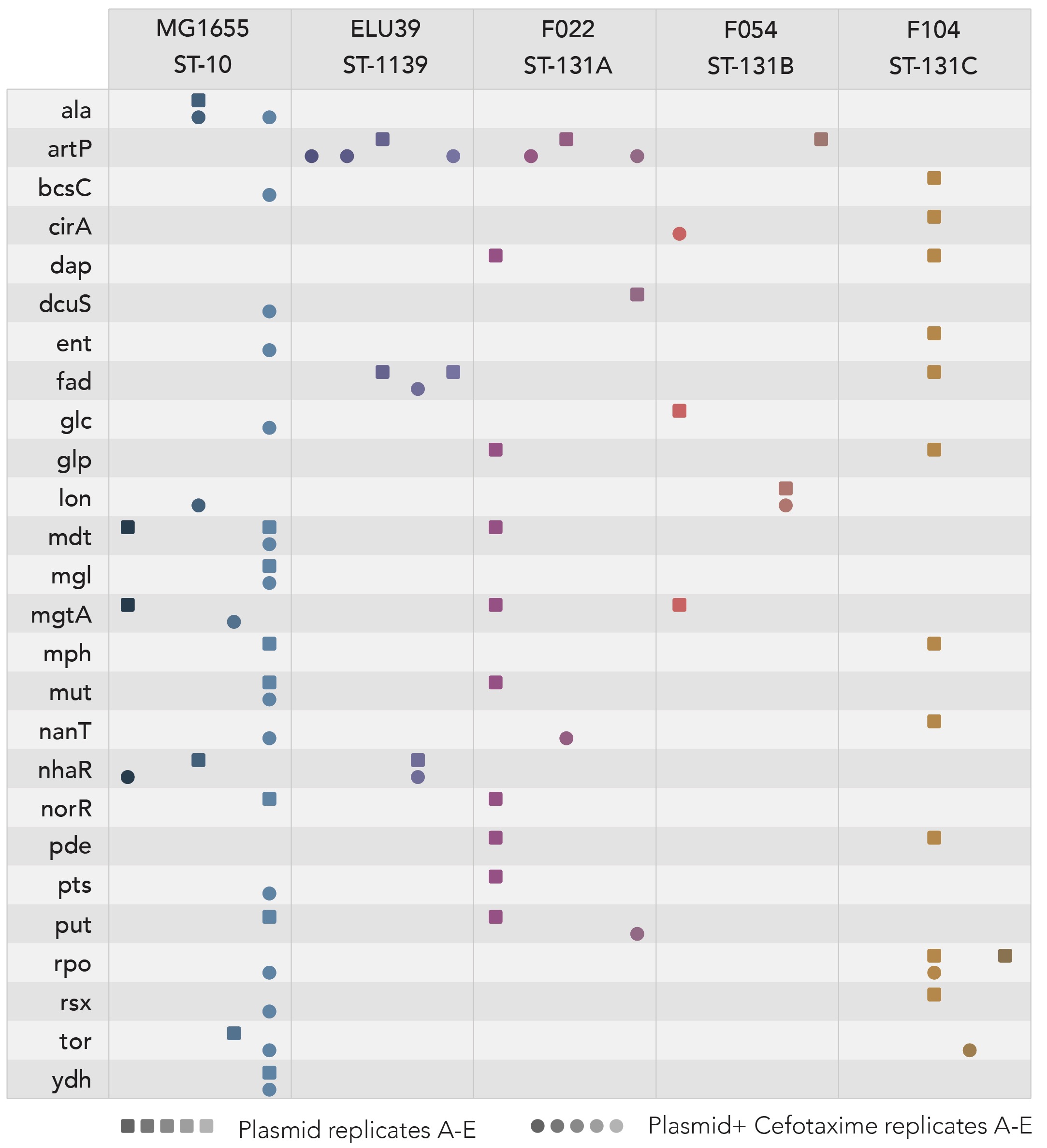

Supplement: FIG S3 [file msystems.00713-22-s0007.jpg]

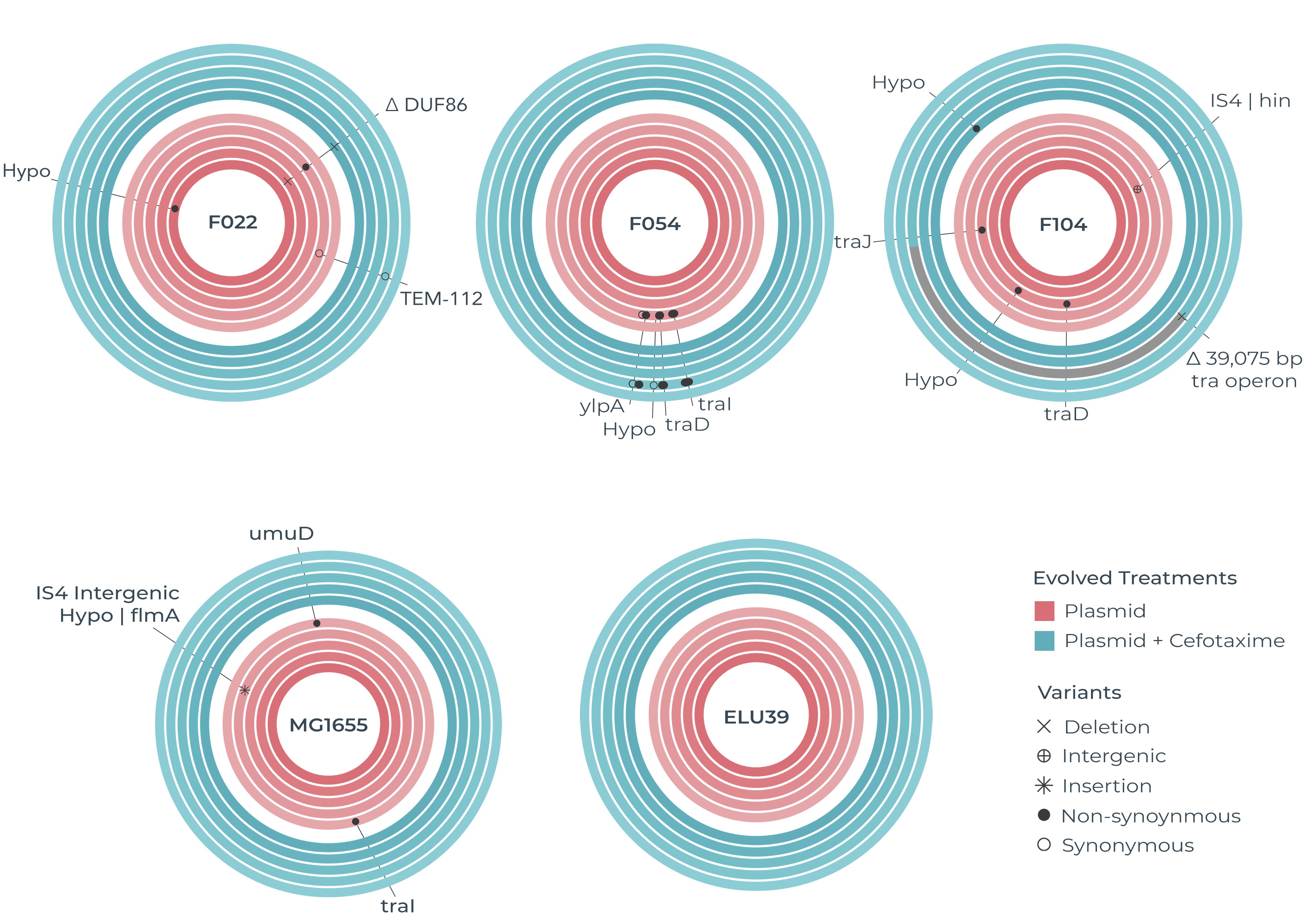

Supplement: FIG S4 [file msystems.00713-22-s0008.jpg]

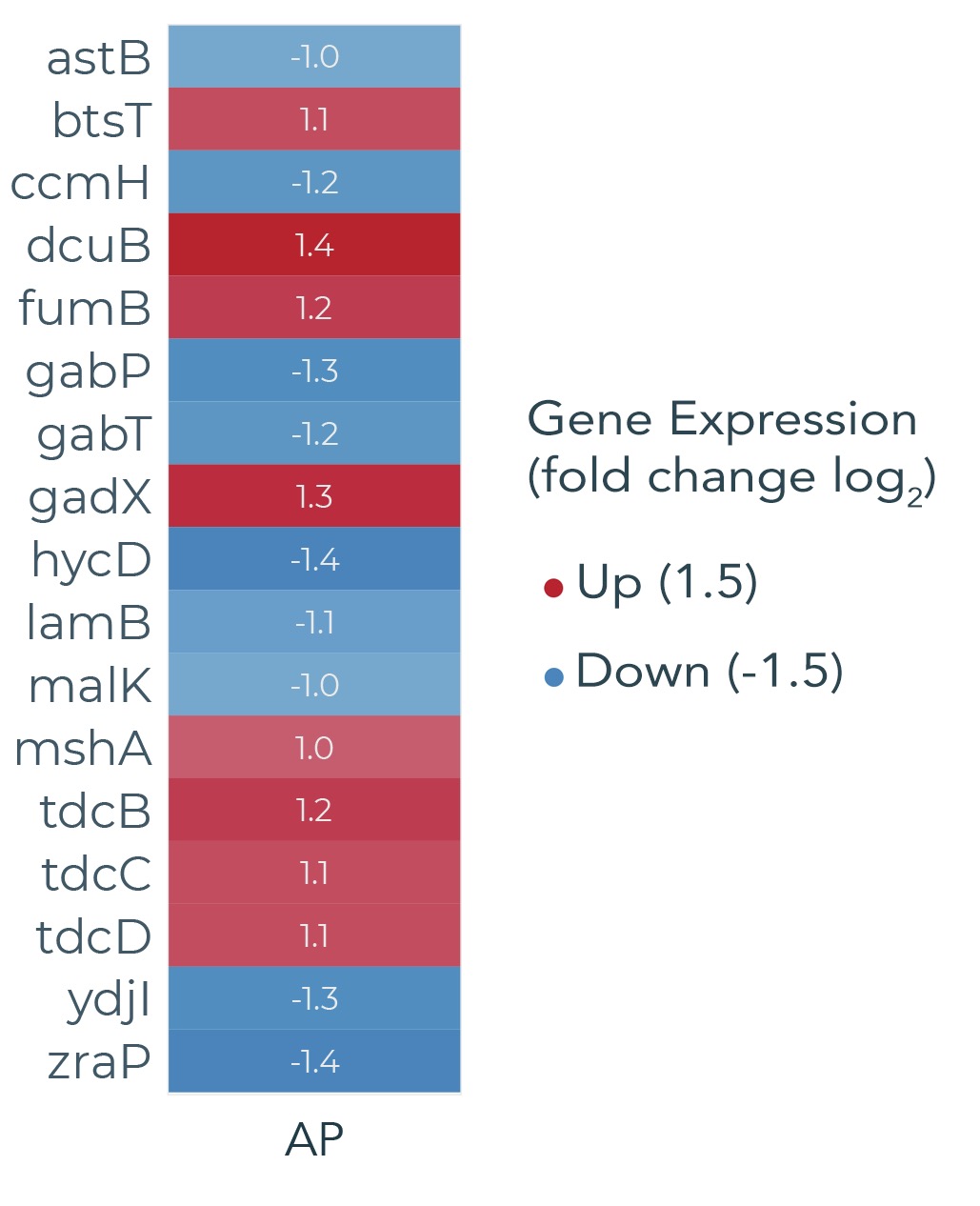

Supplement: FIG S5 [file msystems.00713-22-s0009.jpg]

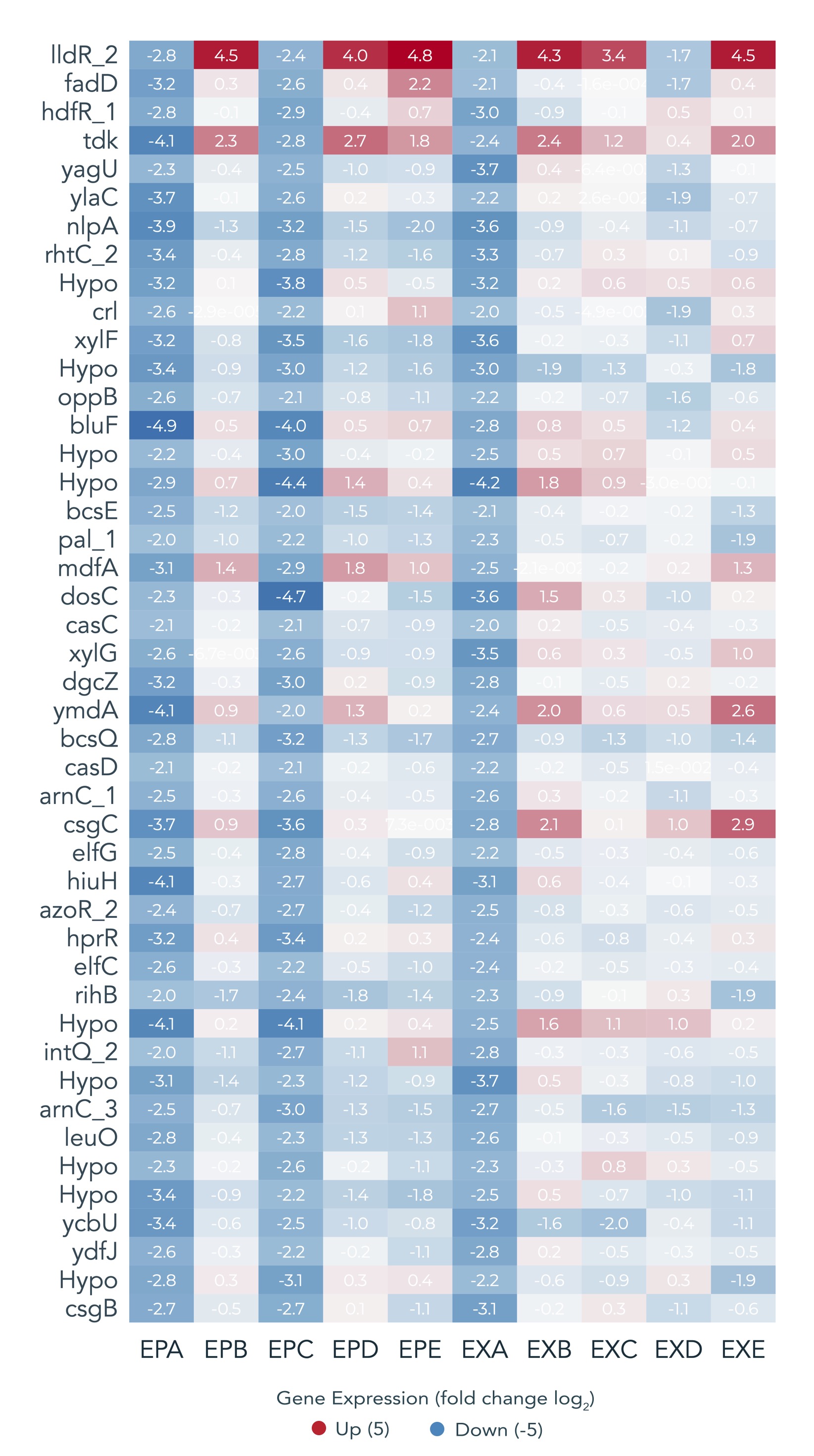

Supplement: FIG S6 [file msystems.00713-22-s0010.jpg]
