# Supplementary material for: Evolutionary Responses to Acquiring a Multidrug Resistance Plasmid Are Dominated by Metabolic Functions across Diverse Escherichia coli Lineages
Source: mSystems. 2023 Feb 1;8(1):e00713-22. doi: 10.1128/msystems.00713-22 (PMC9948715; doi:10.1128/msystems.00713-22)
Supplement: TABLE S3 [file msystems.00713-22-s0002.docx]

| Isolate | Biosample | Illumina | Nanopore |
| --- | --- | --- | --- |
| F022_E0A | SAMN29006941 | SRR19633518 | SRR19634252 |
| F022_E0B | SAMN29006942 | SRR19633517 | SRR19634251 |
| F022_E0C | SAMN29006943 | SRR19633506 | SRR19634240 |
| F022_E0D | SAMN29006944 | SRR19633495 | SRR19634229 |
| F022_E0E | SAMN29006945 | SRR19633484 | SRR19634218 |
| F022_EPA | SAMN29006946 | SRR19633473 | SRR19634207 |
| F022_EPB | SAMN29006947 | SRR19633462 | SRR19634196 |
| F022_EPC | SAMN29006948 | SRR19633451 | SRR19634185 |
| F022_EPD | SAMN29006949 | SRR19633445 | SRR19634179 |
| F022_EPE | SAMN29006950 | SRR19633444 | SRR19634178 |
| F022_EXA | SAMN29006951 | SRR19633516 | SRR19634250 |
| F022_EXB | SAMN29006952 | SRR19633515 | SRR19634249 |
| F022_EXC | SAMN29006953 | SRR19633514 | SRR19634248 |
| F022_EXD | SAMN29006954 | SRR19633513 | SRR19634247 |
| F022_EXE | SAMN29006955 | SRR19633512 | SRR19634246 |
| F054_E0A | SAMN29006956 | SRR19633511 | SRR19634245 |
| F054_E0B | SAMN29006957 | SRR19633510 | SRR19634244 |
| F054_E0C | SAMN29006958 | SRR19633509 | SRR19634243 |
| F054_E0D | SAMN29006959 | SRR19633508 | SRR19634242 |
| F054_E0E | SAMN29006960 | SRR19633507 | SRR19634241 |
| F054_EPA | SAMN29006961 | SRR19633505 | SRR19634239 |
| F054_EPB | SAMN29006962 | SRR19633504 | SRR19634238 |
| F054_EPC | SAMN29006963 | SRR19633503 | SRR19634237 |
| F054_EPD | SAMN29006964 | SRR19633502 | SRR19634236 |
| F054_EPE | SAMN29006965 | SRR19633501 | SRR19634235 |
| F054_EXA | SAMN29006966 | SRR19633500 | SRR19634234 |
| F054_EXB | SAMN29006967 | SRR19633499 | SRR19634233 |
| F054_EXC | SAMN29006968 | SRR19633498 | SRR19634232 |
| F054_EXD | SAMN29006969 | SRR19633497 | SRR19634231 |
| F054_EXE | SAMN29006970 | SRR19633496 | SRR19634230 |
| F104_E0A | SAMN29006971 | SRR19633494 | SRR19634228 |
| F104_E0B | SAMN29006972 | SRR19633493 | SRR19634227 |
| F104_E0C | SAMN29006973 | SRR19633492 | SRR19634226 |
| F104_E0D | SAMN29006974 | SRR19633491 | SRR19634225 |
| F104_E0E | SAMN29006975 | SRR19633490 | SRR19634224 |
| F104_EPA | SAMN29006976 | SRR19633489 | SRR19634223 |
| F104_EPB | SAMN29006977 | SRR19633488 | SRR19634222 |
| F104_EPC | SAMN29006978 | SRR19633487 | SRR19634221 |
| F104_EPD | SAMN29006979 | SRR19633486 | SRR19634220 |
| F104_EPE | SAMN29006980 | SRR19633485 | SRR19634219 |
| F104_EXA | SAMN29006981 | SRR19633483 | SRR19634217 |
| F104_EXB | SAMN29006982 | SRR19633482 | SRR19634216 |
| F104_EXC | SAMN29006983 | SRR19633481 | SRR19634215 |
| F104_EXD | SAMN29006984 | SRR19633480 | SRR19634214 |
| F104_EXE | SAMN29006985 | SRR19633479 | SRR19634213 |
| MG1655_E0A | SAMN29006986 | SRR19633478 | SRR19634212 |
| MG1655_E0B | SAMN29006987 | SRR19633477 | SRR19634211 |
| MG1655_E0C | SAMN29006988 | SRR19633476 | SRR19634210 |
| MG1655_E0D | SAMN29006989 | SRR19633475 | SRR19634209 |
| MG1655_E0E | SAMN29006990 | SRR19633474 | SRR19634208 |
| MG1655_EPA | SAMN29006991 | SRR19633472 | SRR19634206 |
| MG1655_EPB | SAMN29006992 | SRR19633471 | SRR19634205 |
| MG1655_EPC | SAMN29006993 | SRR19633470 | SRR19634204 |
| MG1655_EPD | SAMN29006994 | SRR19633469 | SRR19634203 |
| MG1655_EPE | SAMN29006995 | SRR19633468 | SRR19634202 |
| MG1655_EXA | SAMN29006996 | SRR19633467 | SRR19634201 |
| MG1655_EXB | SAMN29006997 | SRR19633466 | SRR19634200 |
| MG1655_EXC | SAMN29006998 | SRR19633465 | SRR19634199 |
| MG1655_EXD | SAMN29006999 | SRR19633464 | SRR19634198 |
| MG1655_EXE | SAMN29007000 | SRR19633463 | SRR19634197 |
| ELU39_E0A | SAMN29007001 | SRR19633461 | SRR19634195 |
| ELU39_E0B | SAMN29007002 | SRR19633460 | SRR19634194 |
| ELU39_E0C | SAMN29007003 | SRR19633459 | SRR19634193 |
| ELU39_E0D | SAMN29007004 | SRR19633458 | SRR19634192 |
| ELU39_E0E | SAMN29007005 | SRR19633457 | SRR19634191 |
| ELU39_EPA | SAMN29007006 | SRR19633456 | SRR19634190 |
| ELU39_EPB | SAMN29007007 | SRR19633455 | SRR19634189 |
| ELU39_EPC | SAMN29007008 | SRR19633454 | SRR19634188 |
| ELU39_EPD | SAMN29007009 | SRR19633453 | SRR19634187 |
| ELU39_EPE | SAMN29007010 | SRR19633452 | SRR19634186 |
| ELU39_EXA | SAMN29007011 | SRR19633450 | SRR19634184 |
| ELU39_EXB | SAMN29007012 | SRR19633449 | SRR19634183 |
| ELU39_EXC | SAMN29007013 | SRR19633448 | SRR19634182 |
| ELU39_EXD | SAMN29007014 | SRR19633447 | SRR19634181 |
| ELU39_EXE | SAMN29007015 | SRR19633446 | SRR19634180 |

Supplementary Table A – Whole genome sequencing data generated for this work is available under Bioproject Accession number PRJNA848631. Isolate suffixes relate to treatment condition; E0 = Evolved without plasmid, EP = Evolved with plasmid, EX = Evolved with plasmid in the presence of cefotaxime. Ancestral WGS data are available under Bioproject Accession number PRJNA667580, and are described in Dunn *et al.,* 2021 (<https://doi.org/10.1128/mSystems.00083-21>).

| Isolate | Biosample | RNA Replicate 1 | | RNA Replicate 2 | RNA Replicate 3 |
| --- | --- | --- | --- | --- | --- |
| MG1655_A0 | SAMN29009610 | | SRR19635002 | SRR19635000 | SRR19634998 |
| MG1655_AP | SAMN29009611 | | SRR19635001 | SRR19634999 | SRR19634997 |
| MG1655_E0A | SAMN29006986 | | SRR19646225 | SRR19646218 | SRR19646201 |
| MG1655_E0B | SAMN29006987 | | SRR19646224 | SRR19646217 | SRR19646200 |
| MG1655_E0C | SAMN29006988 | | SRR19646213 | SRR19646216 | SRR19646199 |
| MG1655_E0D | SAMN29006989 | | SRR19646202 | SRR19646215 | SRR19646198 |
| MG1655_E0E | SAMN29006990 | | SRR19646191 | SRR19646214 | SRR19646197 |
| MG1655_EPA | SAMN29006991 | | SRR19646185 | SRR19646212 | SRR19646196 |
| MG1655_EPB | SAMN29006992 | | SRR19646184 | SRR19646211 | SRR19646195 |
| MG1655_EPC | SAMN29006993 | | SRR19646183 | SRR19646210 | SRR19646194 |
| MG1655_EPD | SAMN29006994 | | SRR19646182 | SRR19646209 | SRR19646193 |
| MG1655_EPE | SAMN29006995 | | SRR19646181 | SRR19646208 | SRR19646192 |
| MG1655_EXA | SAMN29006996 | | SRR19646223 | SRR19646207 | SRR19646190 |
| MG1655_EXB | SAMN29006997 | | SRR19646222 | SRR19646206 | SRR19646189 |
| MG1655_EXC | SAMN29006998 | | SRR19646221 | SRR19646205 | SRR19646188 |
| MG1655_EXD | SAMN29006999 | | SRR19646220 | SRR19646204 | SRR19646187 |
| MG1655_EXE | SAMN29007000 | | SRR19646219 | SRR19646203 | SRR19646186 |

Supplementary Table B – Accession numbers for all MG1655 transcriptomic data. Samples were sequenced in triplicate, and include ancestral isolate without plasmid (A0), ancestral isolate with plasmid (AP), and experimentally evolved isolates with 5 evolution replicates (A-E), and 3 RNAseq replicates (1-3). E0 = evolved without plasmid, EP = evolved with plasmid, EX = evolved with plasmid in the presence of cefotaxime.
